# A Summary of Current Guidelines and Future Directions for Medical Management and Monitoring of Patients with Cystinuria

**DOI:** 10.3390/healthcare11050674

**Published:** 2023-02-24

**Authors:** Sarah M. Azer, David S. Goldfarb

**Affiliations:** 1 Department of Medicine, NYU Grossman School of Medicine, New York, NY 10016, USA; 2 NYU Langone Medical Center, Nephrology Section, New York Harbor VA Healthcare System, New York, NY 10010, USA

**Keywords:** cystine/metabolism, genetics, kidney calculi, nephrolithiasis, renal aminoaciduria, urolithiasis

## Abstract

Cystinuria is the most common genetic cause of recurrent kidney stones. As the result of a genetic defect in proximal tubular reabsorption of filtered cystine, increased urine levels of the poorly soluble amino acid result in recurrent cystine nephrolithiasis. Recurrent cystine stones not only adversely affect the quality of patients suffering from cystinuria but also may result in chronic kidney disease (CKD) from recurrent renal injury. Thus, the mainstay of medical management revolves around prevention of stones. Recently published consensus statements on guidelines for managing cystinuria were released from both the United States and Europe. The purpose of this review is to summarize guidelines for medical management of patients with cystinuria, to provide new insight into the utility and clinical significance of cystine capacity—an assay for monitoring cystinuria, and to discuss future directions for research on treatment of cystinuria. We discuss future directions, including the potential use of cystine mimetics, gene therapy, V2-receptor blockers, and SGLT2 inhibitors, topics which have not appeared in more recent reviews. It is notable that in the absence of randomized, controlled trials, the recommendations cited here and in the guidelines are based on our best understanding of the disorder’s pathophysiology, observational studies, and clinical experience.

## 1. Introduction

Cystinuria is a rare autosomal recessive genetic disease that impairs reabsorption of cystine and dibasic amino acids (ornithine, lysine, arginine) by the proximal tubule of the kidney and epithelial cells of the gastrointestinal tract. It occurs because of mutations in *SLC3A1* and *SLC7A9* genes that encode the components of the cystine transporter. *SLC7A9* encodes the functional amino acid transporter b^0,+^AT, which transports neutral and dibasic amino acids. *SLC3A1* encodes rBAT, a trafficking protein that helps direct the b^0,+^AT transporter to the apical membrane of proximal tubular epithelial cells. Mutations in either *SLC3A1* or *SLC7A9* lead to defective reabsorption and intra-tubular and urinary accumulation of cystine and dibasic amino acids [1,2]. 

Increased urine levels of poorly soluble cystine are of clinical significance as they result in recurrent cystine nephrolithiasis. While rare, the disorder accounts for 1% of nephrolithiasis in adults and 7% in children [3]. Recurrent cystine stones not only adversely affect the quality of patients suffering from cystinuria but also may result in chronic kidney disease (CKD) from recurrent renal injury [4]. Thus, the mainstay of medical management revolves around prevention of stones. Recently published consensus statements on guidelines for managing cystinuria were released from groups in both the United States and Europe [5,6]. The purpose of this review is to summarize guidelines for medical management of patients with cystinuria, to provide new insight into the utility and clinical significance of cystine capacity—an assay for monitoring cystinuria, and to discuss future directions for research on treatment of cystinuria. Such future directions include the potential use of cystine mimetics, gene therapy, V2-receptor blockers, and SGLT2 inhibitors, topics which have not appeared in more recent reviews. Other reviews which summarize discussions of genetics and pathophysiology are available. We concentrate here on the recent guidelines and newer topics as well [7,8,9].

## 2. Treatment

### 2.1. Conservative Management

Medical management of cystinuria begins with a series of core conservative measures aimed at stone prevention—high fluid intake, minimizing intake of dietary sodium and animal protein, and urinary alkalinization [5,6]. Patients should consume enough fluids to maintain 24-h urine cystine concentrations of less than 250 mg/L (1 mmol/L), usually equating to greater than 3 L urine output each day in adults [10,11]. 

Through a mechanism that is poorly understood, dietary sodium restriction has been shown to decrease levels of cystine in the urine [12,13,14,15]. It is therefore recommended to decrease sodium intake to less than 2.5 g/day [5] or less than 100 mEq of sodium/day by US guidelines, or about 6 g of NaCl/day or 1 to 1.5 mEq/kg, by European guidelines [6]. Restriction of consumption of dietary animal protein also lowers levels of cystine excretion by reducing the intake of cystine and its precursor, methionine. In addition, less animal protein intake reduces the intake of protons, yielding higher urinary pH and increased cystine solubility [10,11,16]. There is no consensus as to how much to restrict animal protein, with suggestions of less than 8 ounces/day [5] and less than 1 g/kg ideal body weight [6]. The former may seem high and the latter low, with the message of both guidelines being that less animal protein is preferred. There are also concerns raised in the pediatric population regarding the effect of animal protein restriction on growth, so significantly limiting animal protein consumption in this population is generally not advised [6,16]. Urinary alkalinization is also recommended to increase urinary cystine solubility [17,18,19,20]. Potassium citrate, with 40–80 mEq per day divided into two or three doses, is recommended as a first-line agent to reach the goal urinary pH of 7.0 or up to 8.0 [5,6]. The European Association of Urology guidelines on urolithiasis suggests that going as high as 8.5 may be appropriate [21]. The guidelines offer slightly different ranges. We aim initially for values greater than 7.0 and further increase if stone growth is persistent. The increased risk of calcium phosphate nephrolithiasis with higher urinary pH is often discussed but rarely seen [6,19]. Alkalinization of the urine increases citrate excretion, which, in turn, inhibits calcium stone formation [22].

We postulate that the lack of randomized control trials leads to the differences in values recommended when comparing both consensus statements. Furthermore, while there are studies that demonstrate decreased urinary cystine levels with alterations of these parameters, there currently are no studies that demonstrate decreased incidence of cystine nephrolithiasis with modification of dietary sodium and animal protein or urinary alkalinization [5,6]. Nevertheless, given the effects on urine chemistry, these are reasonable recommendations, though strict patient adherence to the above parameters is often challenging.

### 2.2. Pharmacotherapy

Therapy for cystinuria should be escalated in a stepwise fashion, with pharmacotherapy with cystine binding thiol drugs (CBTD) added for patients who continue to have recurrent cystine nephrolithiasis despite implementation of and adherence to conservative therapy [5,6]. These drugs, alpha-mercaptopropionylglycine (tiopronin) and D-penicillamine, work through the reduction of the disulfide bond of cystine, yielding a more soluble drug-cysteine complex and reducing free urinary cystine levels [23,24]. The use of CBTDs has been demonstrated to be effective in reducing cystine stone growth, new stone formation, and incidence of urologic intervention in retrospective studies [25,26,27,28,29,30,31,32]. However, their use is limited to cases refractory to conservative therapy given their high cost, adverse effect profile, including drug sensitivity reactions, nephrotic range proteinuria secondary to membranous nephropathy, and very rare liver abnormalities and hematologic disturbances, such as neutropenia and thrombocytopenia [26,33]. Of note, the U.S. Food and Drug Administration (FDA) recently removed a recommendation to monitor complete blood counts and liver function tests when administering tiopronin. Oral vitamin B6 is sometimes recommended for patients on CBTDs to prevent pyridoxine deficiency [5]. Recent data suggest that in vitro tiopronin is more effective at more alkaline pH values. Although this effect has not been demonstrated in people because of a lag in getting urine from the bladder, we recommend that all patients taking CBTDs also remain on alkali regardless of their clinical status [34]. Tiopronin is currently not widely available outside the United States.

Early literature demonstrated decreased incidence of adverse effects with tiopronin compared with penicillamine [29], thus it is generally considered the first-line agent. The starting dose of tiopronin is 15 to 40 mg/kg/day, which generally equates to about 600 to 900 mg daily, divided into three doses [5,6]. No maximal dose is cited. The average dose in clinical trials has been about 800 mg/d. Higher doses may be appropriate if stone formation persists. However, a recent study on 442 patients with cystinuria on CBTDs in France demonstrated a similar incidence of adverse effects with both drugs [19]. The starting dose of D-penicillamine is 20 to 30 mg/kg/day, around 500 to 1500 mg/day in adults, divided into four doses.

Kidney stones, regardless of composition, negatively affect health-related quality of life. Cystine stone formers are more frequent and severe stone formers compared with non-cystine stone formers, resulting in a greater, direct effect on the quality of life in cystinuria [35]. In a recent study, we showed that patients taking tiopronin generally had a better quality of life than patients with cystinuria not taking CBTDs [35].

## 3. Surveillance

Recommended surveillance for patients with cystinuria focuses on monitoring urine and serum chemistries, periodic renal imaging, and screening of family members. Both American and European guidelines recommend obtaining a 24 h urine sample upon initiation of therapy and annually thereafter in patients with stones to monitor the effectiveness of therapies implemented. These samples should be monitored for urine volume, pH, sodium, and creatinine. Spot urine testing on freshly voided samples for pH, the presence of crystalluria, and specific gravity can be helpful in the clinic. Self-monitoring and reporting of at-home measurements of urine pH and specific gravity are also desirable. Measurement of protein/creatinine ratios is appropriate for patients on CBTDs, with history of frequent urologic interventions, or chronic kidney disease (CKD). Monitoring of 24 h urine cystine concentrations is also recommended with the goal concentration of less than 250 mg/L. However, the measurement is consistently noted to have inherent flaws [36,37]. Most frequently reported is the inability of the assay to differentiate between cystine and the cystine-thiol drug complex, lending to inaccurate measurements in patients on CBTDs [38]. However, even in patients not on CBTDs, measurements are still not reliable, given how variable the solubility of urinary cystine is at different pH values. 

A solid phase assay, called cystine capacity, has been proposed as a more reliable method for quantifying urine cystine levels [37]. The assay works through the addition of a known amount of solid cystine to patient urine samples, which are then spun for 48 h at 37 °C, and the crystals harvested. In undersaturated urine, the crystals will dissolve, and the urine is said to have a “positive” capacity as it dissolves more of the solid cystine with less solid cystine recovered. Supersaturated urine has a “negative” capacity as the solid cystine grows, taking cystine up from the urine, with more solid cystine recovered [38]. Studies have demonstrated the accuracy of cystine capacity in the presence of CBTDs [37] and less urinary supersaturation in patients on CBTDs, as demonstrated by more-positive or less-negative capacities [30,38]. Only recent literature has studied cystine capacity relative to stone events. Therefore, its sensitivity and specificity as a predictor of clinical outcomes are uncertain. A recent study on 48 patients with cystinuria demonstrated significantly increased cystine capacity in patients without stone events when compared to patients with stone events, with a strong inverse correlation between cystine capacity and stone activity [39]. Similarly, an ongoing study has preliminary data released on 26 patients with cystinuria enrolled in the Rare Kidney Stone Consortium. Patients with positive cystine capacity were found to have significantly higher urine volumes, lower 24 h urine cystine excretion levels, and higher urine pH. Additionally, patients with positive cystine capacity were found to have a significantly lower incidence of stone events—defined as a development of a new stone, urologic stone removal, and stone passage without intervention [40]. Overall, cystine capacity appears to be a more reliable and accurate way to monitor patients with cystinuria and assess their response to therapy. A recent study demonstrated liquid chromatography/mass spectroscopy may be superior for measurement of cystine-drug complexes [36].

Additionally, both American and European guidelines recommend periodic kidney imaging with either computed tomography (CT) or ultrasonography at least annually in patients with stone events, with a frequency determined on a case-by-case basis. While CT is more sensitive, ultrasonography is generally preferred to minimize exposure to radiation, especially in the pediatric population [5,6].

Screening for cystinuria in siblings of patients with cystinuria is also universally recommended. American guidelines also advise at least yearly measurement of serum creatinine in all patients with cystinuria to monitor for the development of CKD [5]. European guidelines advise that patients self-monitor urinary pH with urine dipsticks, test strips, or electronic devices to ensure adequate alkalinization [6].

## 4. Future Directions

While recent literature on cystinuria has helped shed light on already established therapeutics and methods of surveillance, treatment options have generally not changed over the past few decades. Despite challenges in adherence to conservative measures and pharmacotherapy with CBTDs due to adverse effects, there remains a paucity of clinical trials testing new pharmacotherapeutic options in humans. A recent proposal suggests that glucosuria may interfere with the cystine bond that precipitates cystine stones in the urine. Thus, a phase 2 clinical trial is underway to test the efficacy of dapagliflozin, an inhibitor of the proximal tubule’s sodium-glucose cotransporter 2 (SGLT-2), in patients with cystinuria [41]. Time and further studies will reveal whether dapagliflozin or other SGLT-2 inhibitors may serve as potential treatment options for patients with cystinuria.

Tolvaptan, which blocks the effect of vasopressin by binding to the V2 receptor in the collecting duct, prevented cystine stone growth through increased fluid intake and urine volume in cystinuric mice [42]. A pilot clinical trial is currently underway testing the effect of this aquaretic on cystine capacity in urine from patients with homozygous cystinuria [43]. Another recent study demonstrated that orally administered alpha-lipoic acid inhibited cystine stone growth in *Slc3a1* knockout mice by increasing the solubility of cystine [44]. The mechanism of action of this readily available, over-the-counter supplement is not known. However, a pilot clinical trial is now ongoing to test its effects on cystinuric patients [45]. A suggestion of benefit was offered by a case report on two pediatric patients [46].

An approach that has been proposed as a promising area for new therapeutics to treat cystinuria is gene therapy—a method by which genetic material is changed, removed, or added into human cells to treat the disease [47]. Cystinuria appears to be an ideal candidate for gene therapy given its known and established monogenic basis, the localized expression of defective cystine transporter by cells of the proximal tubule, and the likelihood that even partial reduction in tubular cystine wasting would have a large impact on stone events clinically [47]. Whether this approach could be used for both mutations in *SLC3A1* and *SLC7A9* or how these specific genes will be targeted is not yet known.

Another innovative approach that has demonstrated efficacy for decreasing cystine stone burden in animal models is the inhibition of cystine stone growth through the use of cystine-mimetic agents. The concept was introduced by an in vitro study using atomic force microscopy (AFM) to reveal decreased growth velocity of cystine stones through binding of cystine-mimetic agents, such as cystine dimethyl ester (CDME), to cystine stone surfaces [48]. CDME was thereafter utilized in a study on *Slc3a1* knockout mice, demonstrating decreased stone burden by 50% and small stones formed, but it did not reduce the number of stones [49,50]. These results, while novel and groundbreaking, would be of limited use in humans, given CDME would have poor bioavailability after degradation by intestinal and plasma esterases. In response, Hu et al. designed a series of cystine diamides with greater stability, and thus, bioavailability, with L-cystine bis(*N*′-methylpiperazide) (CDNMP, LH708) in particular demonstrating efficacy in halting stone growth in *Slc3a1* knockout mice [51,52,53]. Additional improvements in diagnostics and therapy could arise from in silico analysis of how specific mutations affect disease pathophysiology [50]. The potential of computational predictions of mutation severity and effects on protein function to correlate with patient phenotypes may also lead to more personalized therapies [51,52],

## 5. Conclusions

Cystinuria is a rare genetic disorder that impairs the cystine transporter from reabsorbing cystine, leading to increased urine cystine levels and cystine nephrolithiasis. Recurrent nephrolithiasis in patients with cystinuria negatively affects the quality of life and may lead to the need for frequent urologic intervention and/or CKD from recurrent kidney injury. Thus, it is important to initiate treatment early and closely monitor patients with cystinuria.

Treatment is centered around stone prevention and escalated in a stepwise manner, starting with conservative measures—high fluid intake, minimizing intake of dietary sodium and animal protein, and urinary alkalinization. In patients with recurrent cystine nephrolithiasis, despite conservative measures, pharmacotherapy with CBTDs may be initiated under close monitoring. Surveillance of patients with cystinuria focuses on interval monitoring of serum and urine chemistries and periodic renal imaging, with specific timing dependent on individual patients’ frequency of stone occurrence. While cystine concentration in 24 h urine samples is still used for monitoring, we highlight numerous inaccuracies with the assay that render it unreliable. Instead, the solid phase assay cystine capacity may provide a more reliable and accurate method to monitor urinary cystine levels, with recent studies demonstrating more positive cystine capacity with a lower incidence of cystine nephrolithiasis.

While there remains an overall paucity of studies testing new therapeutics for cystinuria in humans, a few ongoing human pilot trials are testing the effects of dapagliflozin, tolvaptan, and alpha-lipoic acid on patients with cystinuria. Meanwhile, in vivo studies on knockout mouse models have demonstrated efficacy of molecular imposters in halting cystine stone growth, strongly directing the future of cystinuria therapeutic trials towards this model.

## Data Availability

Not applicable.
